# Identification and validation of *ADME* genes as prognosis and therapy markers for hepatocellular carcinoma patients

**DOI:** 10.1042/BSR20210583

**Published:** 2021-05-27

**Authors:** Jukun Wang, Ke Han, Chao Zhang, Xin Chen, Yu Li, Linzhong Zhu, Tao Luo

**Affiliations:** 1 Department of General Surgery, Xuanwu Hospital of Capital Medical University, Beijing, China; 2 Department of Thoracic Surgery, Xuanwu Hospital of Capital Medical University, Beijing, China

**Keywords:** Hepatocellular carcinoma, prognosis, signature, TCGA

## Abstract

**Purpose**: ADME genes are genes involved in drug absorption, distribution, metabolism, and excretion (ADME). Previous studies report that expression levels of ADME-related genes correlate with prognosis of hepatocellular carcinoma (HCC) patients. However, the role of ADME gene expression on HCC prognosis has not been fully explored. The present study sought to construct a prediction model using ADME-related genes for prognosis of HCC.

**Methods**: Transcriptome and clinical data were retrieved from The Cancer Genome Atlas (TCGA) and International Cancer Genome Consortium (ICGC), which were used as training and validation cohorts, respectively. A prediction model was constructed using univariate Cox regression and Least Absolute Shrinkage and Selection Operator (LASSO) analysis. Patients were divided into high- and low-risk groups based on the median risk score. The predictive ability of the risk signature was estimated through bioinformatics analyses.

**Results:** Six ADME-related genes (*CYP2C9, ABCB6, ABCC5, ADH4, DHRS13*, and *SLCO2A1*) were used to construct the prediction model with a good predictive ability. Univariate and multivariate Cox regression analyses showed the risk signature was an independent predictor of overall survival (OS). A single-sample gene set enrichment analysis (ssGSEA) strategy showed a significant relationship between risk signature and immune status. Gene Ontology (GO) and Kyoto Encyclopedia of Genes and Genomes (KEGG) enrichment analyses showed differentially expressed genes (DEGs) in the high- and low-risk groups were enriched in biological process (BP) associated with metabolic and cell cycle pathways.

**Conclusion:** A prediction model was constructed using six ADME-related genes for prediction of HCC prognosis. This signature can be used to improve HCC diagnosis, treatment, and prognosis in clinical use.

## Introduction

Hepatocellular carcinoma (HCC) is the most common liver cancer type [1]. It is the sixth-leading cause of cancer morbidity and fourth cause of cancer mortality worldwide [2,3]. The main risk factors associated with HCC pathogenesis include hepatitis virus infection, aflatoxin B1 exposure, and alcohol consumption [4]. Prognosis of HCC patients is poor due to high malignancy and rapid progression. Despite advances in treatment approaches, the 5-year survival rate of HCC patients in the United States is only 18% [5]. HCC prognosis and treatment selection is challenging due to its heterogeneity [6]. Therefore, it is necessary to find novel tumor markers and therapeutic targets to improve management of HCC patients.

ADME genes are genes involved in drug absorption, distribution, metabolism, and excretion (ADME). They mainly encode Phase I and II drug-metabolizing enzymes, transporters, and modifiers and are involved in drug metabolism and clearance of drugs by the liver [7]. PharmaADME Consortium identified 298 ADME genes, including 32 core ADME genes and 266 extended ADME genes. In addition, ADME genes are involved in metabolism, transport, and clearance of endogenous and exogenous substances, such as steroid hormones, bile acids, and carcinogens, which can potentially promote cancer initiation [8,9]. Liver diseases, including HCC [10], affect ADME gene expression levels, thus reducing hepatic metabolic capacity and ultimately affecting drug treatment efficacy [7]. Moreover, ADME genes are expressed in extrahepatic and cancer tissues and are correlated with cancer progression and resistance to anticancer drugs [11–13]. Therefore, it is necessary to explore the association between ADME gene expression level and prognosis of cancer patients.

Previous studies have explored the role of ADME genes as prognostic cancer biomarkers and therapeutic targets. Hu et al. [14] reported that approx. half of ADME genes are expressed in 21 cancers and they have prognostic value in these cancers. In addition, most ADME genes are highly expressed in HCC. Moreover, several studies report differential expression of ADME genes in HCC and non-cancerous tissues. Chen et al. [15] reported that CYP1A2 expression is significantly decreased in HCC tissues compared with the level in normal tissues. Similarly, Yan et al. [16] reported that expression levels of the nine CYPs and five UGTs in HCC and paired adjacent non-cancerous tissues are different from the levels in normal tissues. These studies report that ADME genes are prognostic and diagnostic biomarkers in HCC. However, no study has constructed an HCC prediction model using ADME genes.

In the present study, mRNA expression data and corresponding clinical information of HCC patients were retrieved from public databases. The Cancer Genome Atlas (TCGA) cohort was used to construct a prediction model based on ADME-related genes, and its predictive ability was validated using International Cancer Genome Consortium (ICGC) cohort. Furthermore, the relationship between the biological function and ADME-related signature risk score in HCC was explored. The present study provides information for development of therapeutic strategies for HCC patients.

## Materials and methods

### Data collection

RNA-seq data and corresponding clinicopathological data of 371 HCC patients were retrieved from TCGA on 14 February 2021 (https://portal.gdc.cancer.gov/) and was used as the training cohort. Similarly, data for 231 HCC samples were retrieved from ICGC (https://dcc.icgc.org/releases) database (validation cohort). The samples were from Japanese individuals [17]. A set of ADME-related genes (*n*=298) was acquired from previous literature [14].

### Selection of prognostic ADME-related differentially expressed genes in the TCGA cohort

Differentially expressed genes (DEGs) in HCC samples and adjacent nontumorous tissues from TCGA cohort were screened using the R package ‘limma’, with a |log2FoldChange| ≥ 1 and a false discovery rate (FDR) < 0.05. Prognosis-associated ADME-related genes were identified using univariate Cox regression analysis. Venn R package was used to determine genes that overlap for further analysis.

### Construction and validation of a prediction model using ADME-related genes

A prediction model was constructed using Least Absolute Shrinkage and Selection Operator (LASSO) regression analysis by eliminating collinearity in genes using the R package ‘glmnet’. The risk score of each patient was then calculated as follows:

$$\text{Risk score}=\beta_{1}\times {\text{Exp}}_{1}+\;\beta_{2}\times {\text{Exp}}_{2}+\;\beta_{\text{i}}\times {\text{Exp}}_{\text{i}}$$

where β represents the regression coefficient, and Exp represents ADME-related gene expression levels. Patients were divided into high- and low-risk groups based on the median risk score. Moreover, ‘survival’ and ‘survivalROC’ packages were used to generate survival and receiver operating characteristic (ROC) curves to estimate the accuracy of the prediction model. Principal component analysis (PCA) and T-distributed Stochastic Neighbor Embedding (t-SNE) analysis were performed using ‘prcomp’ and ‘Rtsne’ packages to determine the distribution of the genes in different groups. In addition, 231 HCC samples from ICGC database were used to validate the predictive ability of the prediction model.

### ADME-related risk signature score and other clinicopathological features

ADME-related risk signature score was compared with conventional clinicopathological characteristics using univariate and multivariate Cox regression analyses to determine if it was an independent prognostic factor for overall survival (OS).

### Single-sample gene set enrichment analysis

Levels of 16 types of infiltrating immune cells and activity of 13 immune-related pathways or functions were determined using the R package ‘gsva’ through single-sample gene set enrichment analysis (ssGSEA).

### Functional enrichment analysis

The R package ‘clusterProfiler’ was used to perform Gene Ontology (GO) and Kyoto Encyclopedia of Genes and Genomes (KEGG) analyses using DEGs in the high- and low-risk groups. DEGs were selected using |log2FoldChange| ≥ 1 and FDR < 0.05. GO analysis included biological processes (BPs), cellular components (CCs), and molecular functions (MFs).

### Statistical analyses

R software (version 4.0.2) was used for all statistical analyses. DEGs in tumor tissues and adjacent non-tumor tissues were identified using Wilcoxon test. Mann–Whitney test was used to compare ssGSEA scores of immune cells or pathways between the high- and low-risk groups. Survival time in the high- and low-risk groups was estimated using Kaplan–Meier curves. In addition, log-rank test was used to analyze differences in survival time. ROC curves and corresponding area under the ROC curve (AUC) values were used to evaluate accuracy of the ADME-related risk signature score. Independent predictors of OS were identified using univariate and multivariate Cox regression analyses. *P*-values less than 0.05 were considered statistically significant, and all *P*- values were two-tailed.

## Results

### Identification of prognostic ADME-related DEGs in the TCGA cohort

**Figure 1:**
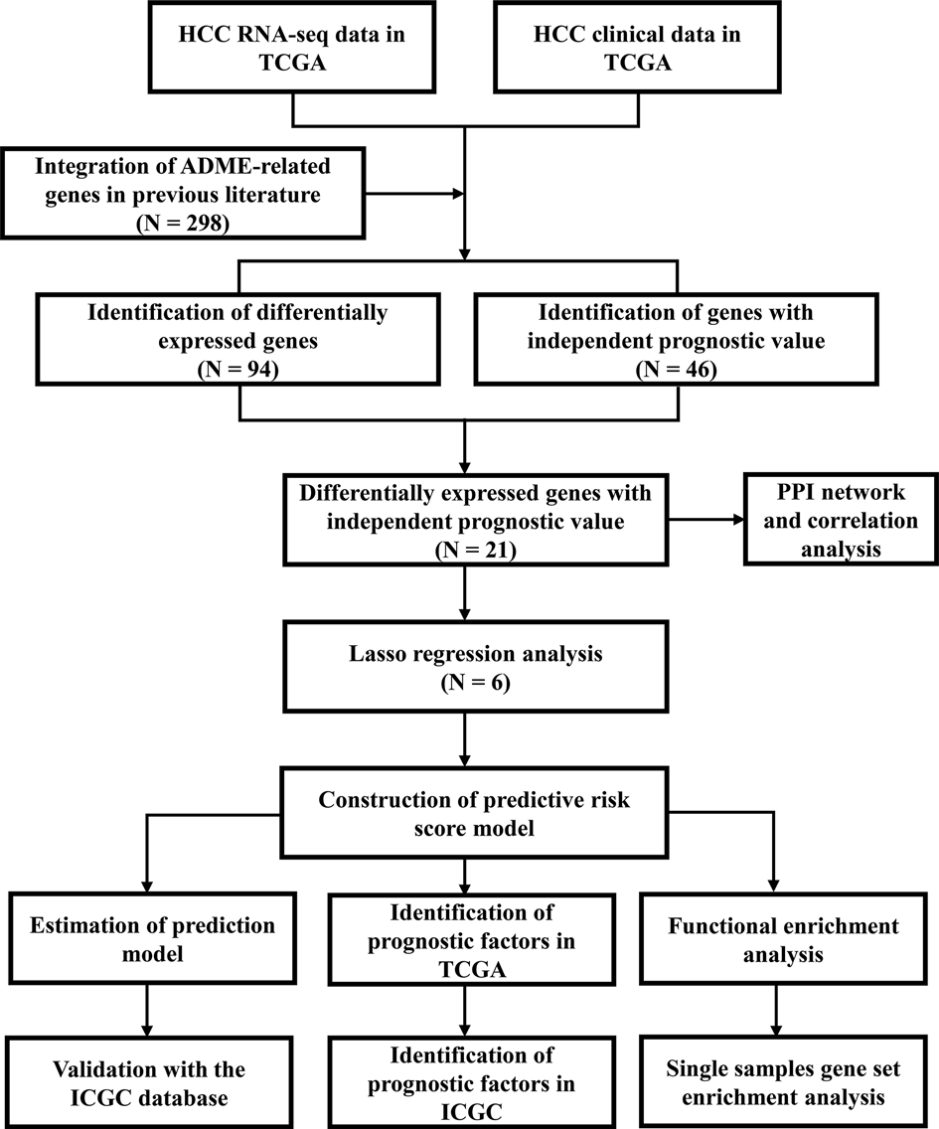
Flow chart of data analysis

**Figure 2:**
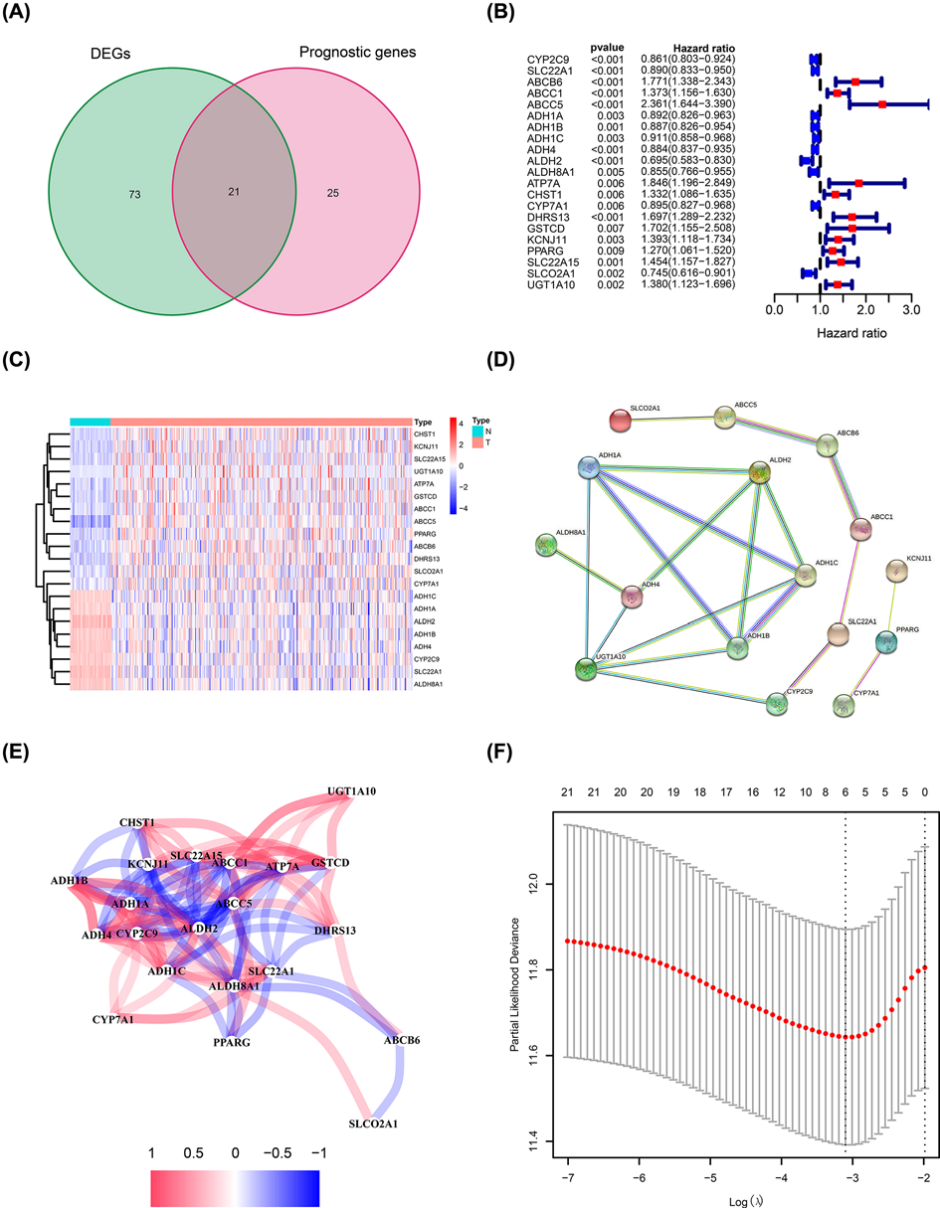
Prediction model construction using ADME-related genes (**A**) Venn plot showing intersection of genes related to OS and differentially expressed in tumor and adjacent normal tissues. (**B**) Forest plots showing the relationship between expression of intersecting genes and OS. (**C**) Heatmap showing relative expression of intersecting genes in tumor and adjacent normal tissues. (**D**) PPI network of intersecting genes. (**E**) Correlation analysis of intersecting genes. (**F**) Prediction model construction using LASSO regression analysis.

The study flow chart is shown in Figure 1. Data of 365 HCC patients from the TCGA cohort were used as the training dataset except for six patients (the follow-up of five patients was not conducted and one patient opted out of the study). Data for 231 HCC patients from the ICGC cohort were used as validation datasets. Univariate Cox regression analysis showed that 46 ADME-related genes were significantly correlated with OS (*P*<0.01). Out of the 46 genes, 21 genes were differentially expressed in cancerous tissues compared with adjacent non-cancerous tissues (Figure 2A,B). A heatmap was used to visualize differential expression levels of the 21 genes (Figure 2C). Protein–protein interaction (PPI) network analysis and correlation analysis were used to explore the interactions among the 21 genes (Figure 2D,E).

### Prediction model construction using the TCGA cohort

**Figure 3:**
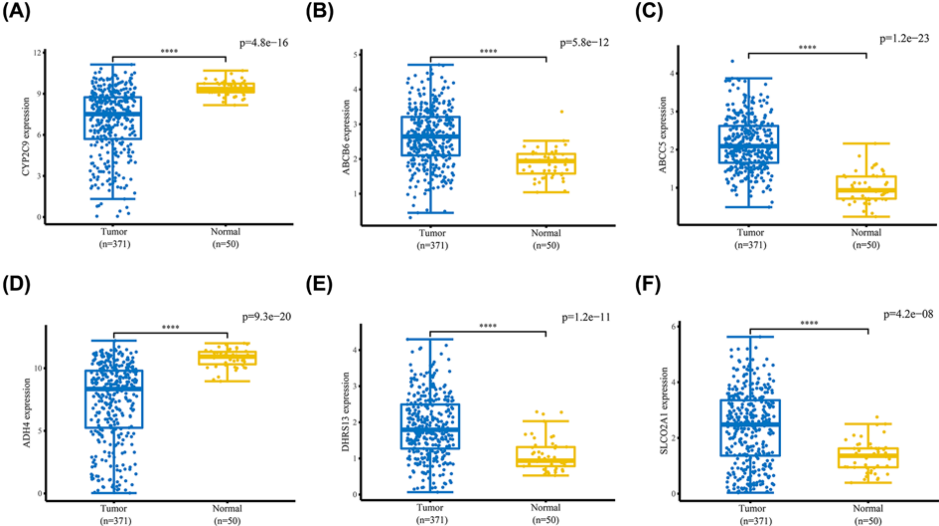
Expression levels of the six ADME-related genes between HCC and adjacent normal tissues mRNA expression levels of (**A**) CYP2C9 and (**D**) ADH4 were significantly lower in HCC tissues compared with those in adjacent normal tissues, whereas mRNA expression levels of (**B**) ABCB6, (**C**) ABCC5, (**E**) DHRS13, and (**F**) SLCO2A1 were significantly higher in HCC tissues compared with adjacent normal tissues. ****P<0.0001.

LASSO regression analysis was used for construction of the prediction model to minimize risk of overfitting. Six genes (including *CYP2C9, ABCB6, ABCC5, ADH4, DHRS13*, and *SLCO2A1*) were chosen for the construction of the prediction model based on LASSO regression analysis results (Figure 2F). Differential expression levels of the six genes between tumor tissues and adjacent normal tissues are shown in Figure 3. The risk score of each sample was calculated as follows:

$$\begin{array}{l} \text{Risk}\;\text{score}=(-0.060\times \text{CYP}2\text{C}9)+(0.277\times \text{ABCB}6)+(0.236\times \text{ABCC}5)+(-0.041\times \text{ADH}4)\\ +(0.204\times \text{DHRS}13)+(-0.008\times \text{SLCO}2\text{A}1) \end{array}$$

### Prediction model evaluation using TCGA cohort

**Figure 4:**
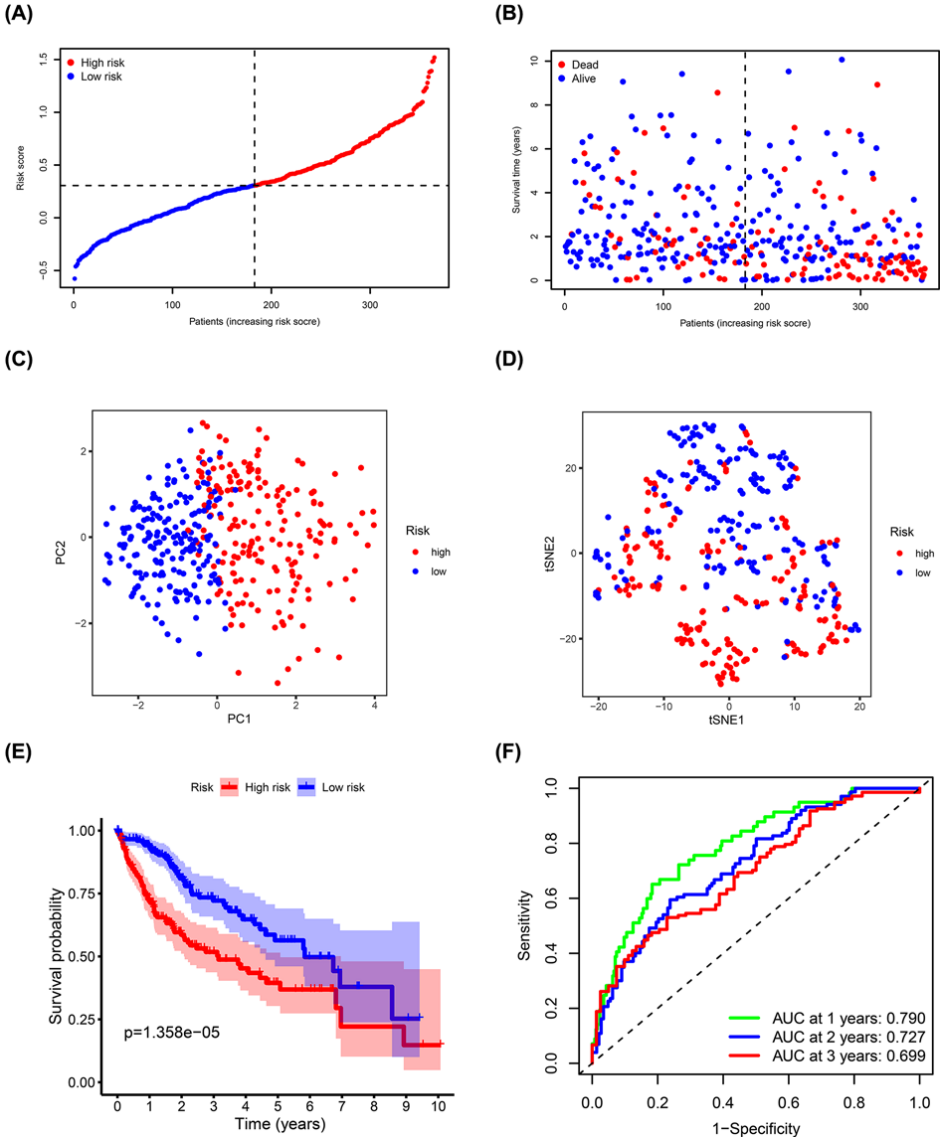
Estimation of ADME-related risk signature using the TCGA cohort (**A**) Distribution of risk scores. (**B**) Distribution of survival status, survival time, and risk scores. (**C**) PCA of risk scores. (**D**) t-SNE analysis of risk scores. (**E**) Kaplan–Meier curves for OS of patients in the high- and low-risk groups. (**F**) ROC curves and AUCs showing the predictive performance of the risk signature.

TCGA cohort samples were divided into high- (*n*=182) and low-risk (*n*=183) groups based on the median risk score and were used to evaluate the predictive ability of the model (Figure 4A). Risk plot showed that high-risk score patients had a shorter survival time compared with the low-risk score patients (Figure 4B). Moreover, PCA and t-SNE analyses showed that the different groups had distinct layout modes (Figure 4C,D). Furthermore, Kaplan–Meier analysis showed that the high-risk group had poorer OS compared with the low-risk group (*P*=1.358e-05) (Figure 4E). AUC values of the risk signature scores were 0.790 at 1 year, 0.727 at 2 years, and 0.699 at 3 years (Figure 4F).

### Prediction model validation using ICGC cohort

**Figure 5:**
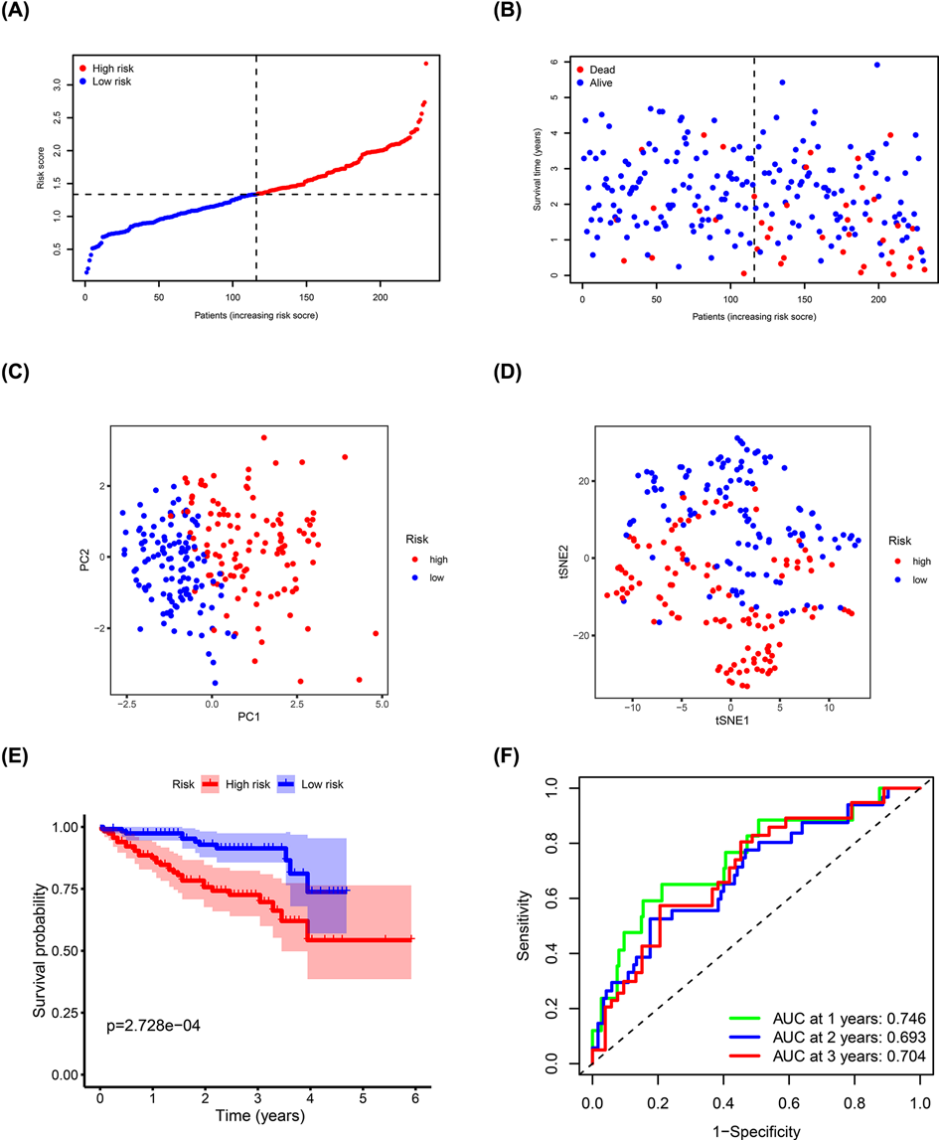
Validation of ADME-related risk signature using the ICGC cohort (**A**) Distribution of risk scores. (**B**) Distribution of survival status, survival time, and risk scores. (**C**) PCA of risk scores. (**D**) t-SNE analysis of risk scores. (**E**) Kaplan–Meier curves for OS of patients in the high- and low-risk groups. (**F**) ROC curves and AUCs showing predictive performance of the risk signature.

ICGC cohort samples were used as the validation dataset to test the performance of the prediction model. ICGC cohort samples were divided into high- (*n*=115) and low-risk (*n*=116) groups using the formula for the TCGA cohort (Figure 5A). Risk plot showed that high-risk score patients had shorter survival compared with that of the low-risk score patients, which was consistent with TCGA cohort results (Figure 5B). Dimensionality reduction analysis using PCA and t-SNE showed that patients in different risk groups were distributed in two directions (Figure 5C,D). Kaplan–Meier analysis confirmed that high risk score patients had a poorer prognosis compared with those with low-risk scores (*P*=2.728e-04; Figure 5E). AUC values of the risk signature scores were 0.746 at 1 year, 0.693 at 2 years, and 0.704 at 3 years (Figure 5F).

### Independent prognostic role of the ADME-related risk signature score using TCGA and ICGC cohorts

**Figure 6:**
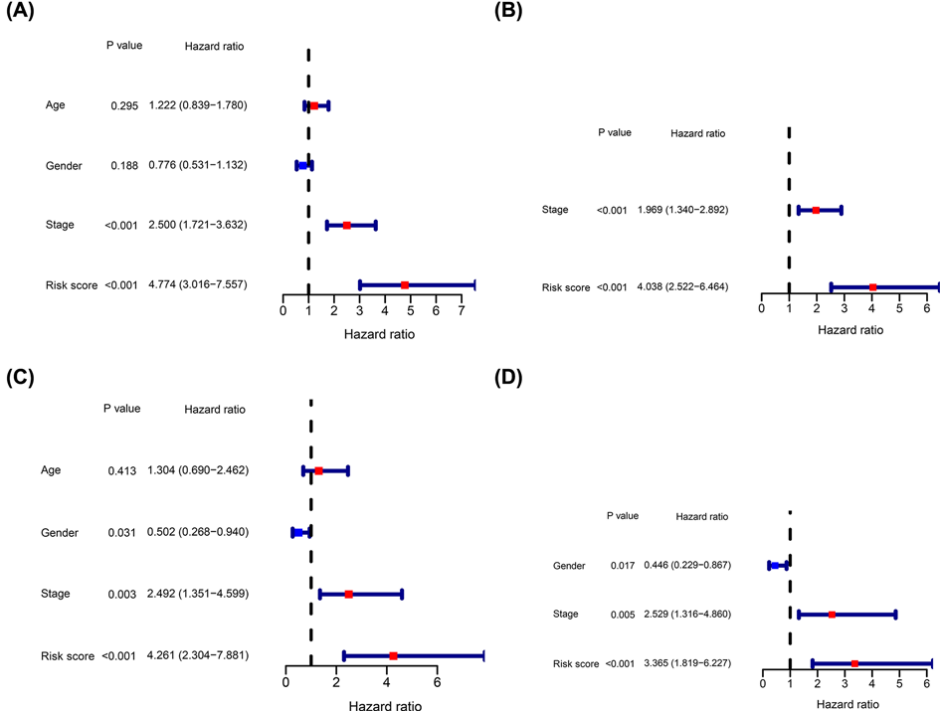
Estimation of the independent prognostic value of the risk signature Univariate Cox regression analysis of OS for HCC patients using the TCGA (**A**) and ICGC (**C**) cohorts. Multivariate Cox regression analysis of OS for HCC patients using the TCGA (**B**) and ICGC (**D**) cohorts.

The independent prognostic value of the ADME-related risk signature score for OS in TCGA and ICGC cohorts was determined using univariate and multivariate Cox regression analyses. Univariate Cox regression analysis showed that the risk score was an independent factor in the TCGA cohort (Figure 6A). Similarly, multivariate Cox regression analysis showed that the risk score was an independent predictor of OS (Figure 6B). In addition, analysis showed that the risk score was an independent predictor of OS in the ICGC validation cohort (Figure 6C,D).

### Functional enrichment analysis of DEGs in the high- and low-risk groups

**Figure 7:**
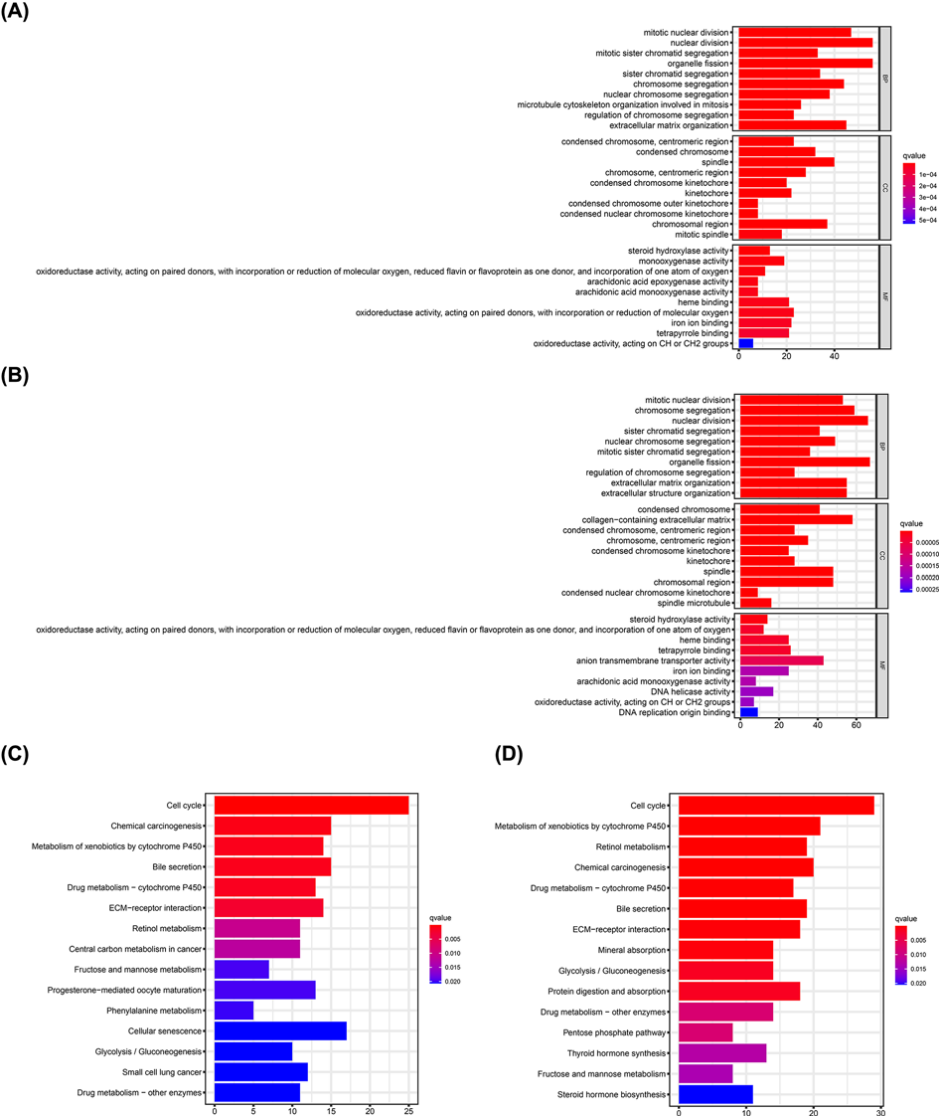
GO and KEGG pathway enrichment analyses GO enrichment analysis of DEGs in the high- and low-risk groups in the TCGA (**A**) and ICGC (**B**) cohorts. KEGG pathway analysis of DEGs in the high- and low-risk groups in the TCGA (**C**) and ICGC (**D**) cohorts.

GO and KEGG pathway enrichment analyses were performed explore the relationship between biological functions of DEGs in the high- and the low-risk groups and ADME-related risk score. GO analysis showed that DEGs were enriched in several metabolism-related MFs, such as steroid hydroxylase, oxidoreductase, and arachidonic acid monooxygenase activities, heme binding, iron ion binding, tetrapyrrole binding, and oxidoreductase activity in both TCGA and ICGC cohorts (Figure 7A,B). Similarly, KEGG analysis showed that DEGs in TCGA and ICGC cohorts were implicated in metabolism-related pathways, such as metabolism of xenobiotic metabolism through cytochrome P450, retinol metabolism, fructose, and mannose metabolism, glycolysis/gluconeogenesis, and drug metabolism (Figure 7C,D). Moreover, DEGs in both TCGA and ICGC cohorts were enriched in BPs involved in cell cycle, such as mitotic nuclear division, nuclear division, organelle fission, sister chromatid segregation, and chromosome segregation regulation.

### Analysis of differences in immune status between the high- and low-risk groups

**Figure 8:**
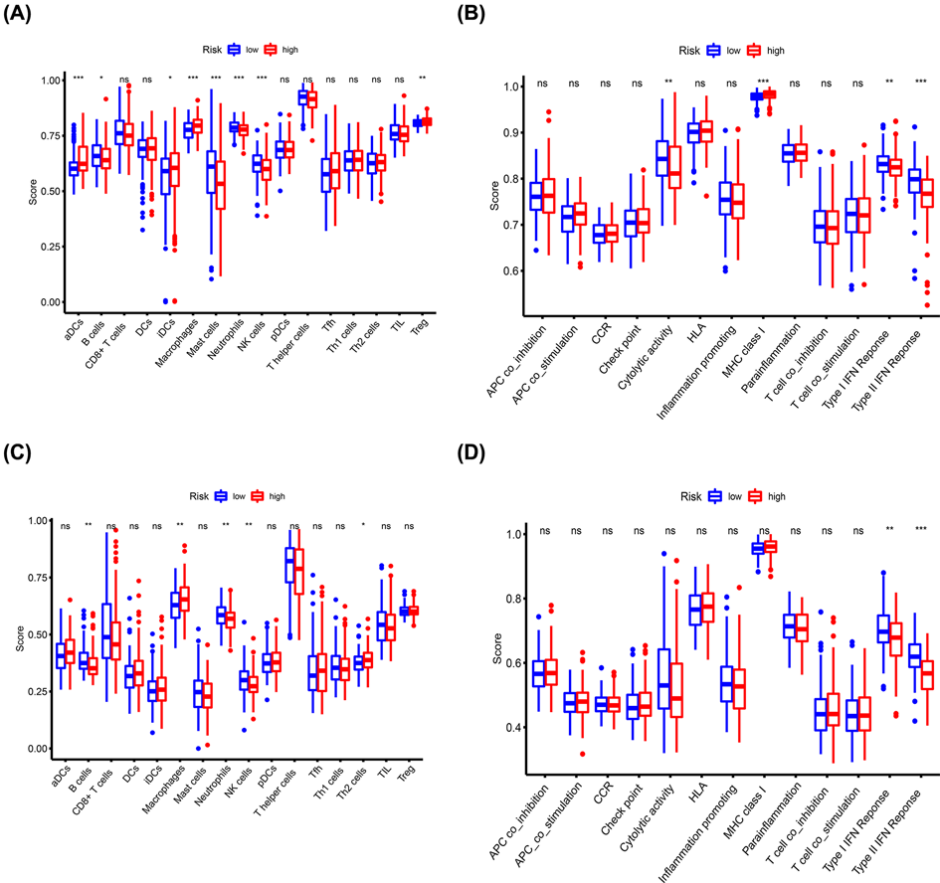
Comparison of the enrichment scores for immune status between the high- and low-risk groups based on ssGSEA Scores of 16 immune cells in the TCGA (**A**) and ICGC (**C**) cohorts. Scores of 13 immune-related functions or pathways in the TCGA (**B**) and ICGC (**D**) cohorts. Abbreviation: CCR, cytokine–cytokine receptor. *P<0.05, **P<0.01, ***P<0.001.

Enrichment scores of immune cell subtypes and immune-related functions and pathways were determined by ssGSEA to explore the correlation between the risk signature score and immune status. Scores of activated dendritic cells (aDCs), inhibited dendritic cells (iDCs), macrophages, regulatory T (Treg) cells, and pathway activations related to major histocompatibility complex (MHC) class I were higher in the high-risk group compared with the scores in the low-risk group. On the contrary, scores of B cells, mast cells, neutrophils, natural killer (NK) cells and pathways related to cytolytic activity, type I interferon (IFN) response, type II IFN response were higher in the low-risk group compared with the high-risk group (Figure 8A,B). Moreover, validation using the ICGC cohort showed that macrophage scores were higher in the high-risk group compared with the scores in the low-risk group, whereas B cells, neutrophils, and NK cell scores and pathways related to the type I IFN response and type II IFN response were higher in the low-risk group compared with the high-risk group (Figure 8C,D).

## Discussion

HCC is a common malignant tumor with complex pathogenesis and high mortality thus it poses a significant threat to global public health. Although significant advances in therapeutic strategies have been achieved, HCC prognosis remains poor, mainly due to late diagnosis and poor response to conventional treatment. Recent studies report multiple molecular markers for prediction model construction in multiple cancers. In addition, these markers have been used for development of therapeutic targets through high-throughput sequencing technology, thus greatly improving early diagnosis and long-term survival of patients.

In the present study, a prediction model was constructed using six ADME-related genes including, *CYP2C9, ABCB6, ABCC5, ADH4, DHRS13*, and *SLCO2A1. CYP2C9*, encodes a member of cytochrome P450 superfamily of enzymes, and is down-regulated in HCC tissues, and patients with low CYP2C9 expression have poor OS and disease-free survival [18,19]. *ABCB6* is a mitochondrial transporter that regulates porphyrin biosynthesis and is up-regulated in HCC tissues. *ABCB6* overexpression enhances HCC cell proliferation and tumorigenicity by targeting the cell cycle [20]. *ABCC5* is a member of the MRP subfamily implicated in transport of various molecules across extra- and intra-cellular membranes [21]. It is associated with multidrug resistance in HCC. Wei et al. reported that *ADH4* is significantly down-regulated in HCC tissues compared with adjacent non-cancerous tissues [22]. In addition, lower *ADH4* expression is significantly correlated with higher pathology grade and poor OS. *SLCO2A1* (OATP2A1) encodes organic anion transporters and is highly expressed in primary and metastatic HCC [23]. High *SLCO2A1* expression level is associated with a poor prognosis [24]. The findings of the current study were consistent with findings from previous studies except for *DHRS13* since no study has explored its role in HCC.

Tumor node metastasis (TNM) staging system is widely used in clinical practice to guide cancer treatment because it is simple and provides clinicians and patients with vital prognostic determinants. However, TNM staging system has some pitfalls. For instance, patients at the same stage may have a different prognosis since the stage system was based on the anatomy of tumor invasion and did not consider the functional status of tumor cells or the patient body [25]. Therefore, this staging system does not fully reflect intratumor heterogeneity, result in ineffective treatment. Therefore, it is essential to screen novel molecular biomarkers to supplement TNM staging for HCC diagnosis and treatment. HCC patients can be divided into high- and low-risk groups based on the median of hypoxia-related risk score [26]. High-risk score patients have a poor prognosis. Several studies have identified novel molecular biomarkers, such as ferroptosis-related genes [27], immune-related genes [28], and metabolism-related genes [29] as prognostic markers and therapeutic targets in HCC. Moreover, the National Comprehensive Cancer Network (NCCN) recommends a 21-gene expression assay (Oncotype DX, Genomics Health) for assessing the prognosis of patients with hormone receptor-positive breast cancer [30]. In this study, a systematic ADME-related genes were analyzed to explore HCC pathogenesis and provide a basis for identification of novel drug targets.

Functional enrichment analysis showed that DEGs in high- and low-risk groups were significantly associated with metabolism-related pathways. A previous study reported that cytochrome P450 is significantly down-regulated in HCC tissues compared with the adjacent non-cancerous tissue. and its expression is correlated with the histological grade of the tumors [31]. Liu et al. [32] also reported that the risk signature of cytochrome P450 can be used for prognosis of HCC patients. Besides, Hu et al. [33] reported that glycolysis plays an important role in HCC progression by providing sufficient energy to meet energy demands for the rapidly proliferating HCC cells. Findings in the current study show that DEGs in different groups were enriched in pathways associated with the cell cycle. Polireddy et al. [34] reported that attenuation of *ABCB6* expression delays G_2_/M phase of the cell cycle, whereas *ABCB6* overexpression promotes HCC cell growth and proliferation.

Tumor immune microenvironment (TIME) is plays important roles in HCC initiation and progression. ssGSEA showed that macrophage enrichment score was higher in the high-risk group compared with the score in the low-risk group in both TCGA and ICGC cohorts. This finding is consistent with finding from a previous study that an increased number of macrophages in the TIME is positively correlated with HCC progression and poor prognosis [35]. Furthermore, the high-risk group had higher Treg cell infiltration compared with the low-risk group. Several studies have explored the immunosuppressive role of the Treg cells in the TIME. Treg cells are implicated in promotion of immune escape of cancer cells through contact-dependent interactions between check-point molecules and their ligands [36]. In addition, Treg cells enhance immunosuppressive environment by releasing inhibitory cytokines [37,38]. Therefore, understanding key mechanisms of modulation of immune escape and immunosuppression by ADME-related genes may provide new strategies for immunotherapy.

To the best of our knowledge, this is the first study to construct a prediction model using ADME-related genes for HCC prognosis. An external validation cohort was used to test the accuracy of the model. In addition, this risk signature can be used to explore the functional states of immune cells and immune signaling pathways. ADME genes can be used as novel biomarkers and targets for HCC diagnosis and treatment. However, this study has some limitations. First, the results should be validated using a large-scale, prospective study since the data used in the present study were retrospectively obtained from public databases. Second, inadequate treatment information, including surgery, ablation, TACE, and target therapy, may reduce the statistical reliability of the findings from the present study. In addition, these findings are based on bioinformatics analysis, therefore, additional validation should be carried out through experimental studies. ADME genes used to construct the prediction model were selected from genes that were differentially expressed between tumor tissues and adjacent normal tissues, and the study did not consider that expression of ADME genes can be affected by different inducers of HCC, including hepatitis viruses B, C, liver cirrhosis, and alcohol liver disease.

## Conclusion

In summary, six ADME-related genes were used for prediction model construction to estimate prognosis of HCC patients. The ADME-related risk signature showed strong predictive ability and was an independent predictor of OS in HCC patients. Therefore, the six ADME-related genes are potential targets for HCC immunotherapy.

## Data Availability

All clinical pathological data of training cohort can be downloaded directly from the TCGA database at: https://portal.gdc.cancer.gov/. The data of validation cohort can be obtained directly from the ICGC database at: https://dcc.icgc.org/releases. In addition, all codes used for data analysis are available from the corresponding author on responsible request.

## Abbreviations

ADME: absorption, distribution, metabolism, and excretion
AUC: area under the ROC curve
BP: biological process
CC: cellular component
DEG: differentially expressed gene
FDR: false discovery rate
GO: gene ontology
HCC: hepatocellular carcinoma
ICGC: International Cancer Genome Consortium
IFN: interferon
KEGG: Kyoto Encyclopedia of Genes and Genomes
LASSO: Least Absolute Shrinkage and Selection Operator
MF: molecular function
NK: natural killer
OS: overall survival
PCA: principal component analysis
ROC: receiver operating characteristic
ssGSEA: single-sample gene set enrichment analysis
t-SNE: T-distributed Stochastic Neighbor Embedding
TCGA: The Cancer Genome Atlas
TIME: tumor immune microenvironment
TNM: tumor node metastasis
Treg: regulatory T
